# A scoping review of complex systems methods used in population physical activity research: do they align with attributes of a whole system approach?

**DOI:** 10.1186/s12961-023-00961-3

**Published:** 2023-03-02

**Authors:** Lori Baugh Littlejohns, Erin Near, Geoff McKee, Drona Rasali, Daniel Naiman, Guy Faulkner

**Affiliations:** 1 BC Centre for Disease Control, 655 W 12th Ave, Vancouver, BC V5Z 4R4 Canada; 2 School of Kinesiology, University of British Columbia, 210-6081 University Boulevard, Vancouver, BC V6T 1Z1 Canada; 3 Department of Population Medicine, University of Guelph, Stewart Building, Building #45, Rm 2509, Guelph, ON N1G 2W1 Canada; 4 School of Population and Public Health, University of British Columbia, 2206 E Mall, Vancouver, BC V6T 1Z3 Canada; 5 BC Ministry of Health, Stn Prov Govt, PO Box 9646, Victoria, BC V8W 9P1 Canada

**Keywords:** Public health research, Complex systems methods, Population physical activity, Whole systems approach

## Abstract

**Background:**

Complex systems approaches are increasingly used in health promotion and noncommunicable disease prevention research, policy and practice. Questions emerge as to the best ways to take a complex systems approach, specifically with respect to population physical activity (PA). Using an Attributes Model is one way to understand complex systems. We aimed to examine the types of complex systems methods used in current PA research and identify what methods align with a whole system approach as reflected by an Attributes Model.

**Methods:**

A scoping review was conducted and two databases were searched. Twenty-five articles were selected and data analysis was based upon the following: the complex systems research methods used, research aims, if participatory methods were used and evidence of discussion regarding attributes of systems.

**Results:**

There were three groups of methods used: system mapping, simulation modelling and network analysis. System mapping methods appeared to align best with a whole system approach to PA promotion because they largely aimed to understand complex systems, examined interactions and feedback among variables, and used participatory methods. Most of these articles focused on PA (as opposed to integrated studies). Simulation modelling methods were largely focused on examining complex problems and identifying interventions. These methods did not generally focus on PA or use participatory methods. While network analysis articles focused on examining complex systems and identifying interventions, they did not focus on PA nor use participatory methods. All attributes were discussed in some way in the articles. Attributes were explicitly reported on in terms of findings or were part of discussion and conclusion sections. System mapping methods appear to be well aligned with a whole system approach because these methods addressed all attributes in some way. We did not find this pattern with other methods.

**Conclusions:**

Future research using complex systems methods may benefit from applying the Attributes Model in conjunction with system mapping methods. Simulation modelling and network analysis methods are seen as complementary and could be used when system mapping methods identify priorities for further investigation (e.g. what interventions to implement or how densely connected relationships are in systems).

**Supplementary Information:**

The online version contains supplementary material available at 10.1186/s12961-023-00961-3.

## Background

Complex systems approaches are increasingly being used in public health, health promotion and noncommunicable disease (NCD) prevention research, policy and practice [1–3]. These approaches answer the call to incorporate holistic systems views and complement reductionist and linear cause–effect approaches [4, 5]. Holistic or whole systems approaches can be described as focusing on ‘people, processes, activities, settings and structures – and the dynamic relationships between them’ [6] (p. 2). Calenbuhr [7] explains that taking a whole systems approach means phenomena such as emergent properties, evolutionary system change and collective decision making are central and requires a shift from the study of parts to the whole system. Hundreds of complex systems methods and approaches have been developed since at least the 1940s, and new innovations are rapidly emerging [8].

The impetus to adopt complex systems approaches is rooted in many factors. For example, reductionist and linear cause and effect approaches often do not adequately reflect conceptualizations of socio-ecological models that are foundational to health promotion and NCD prevention [9]. McLaren and Hawe [10] describe an ecological approach in terms of examining nested circles, where each circle represents a level of influence (i.e. individual, organizational, community, societal). They discuss ecological analysis as focused on the interdependence and interaction among these levels of influence (e.g. how organizational, community and societal factors influence individual health and wellbeing). While socio-ecological approaches are long called for [11], much of the research (and policy and practice) remains focused at the individual level (e.g. lifestyle behavioural change) and/or on specific settings (e.g. communities), with less consideration of systems change at the societal level [12, 13].

Furthermore, health promotion and NCD prevention research, policy and practice has long advocated the need to engage with diverse perspectives (e.g. multiple sectors and levels) to facilitate systems change [14]. For example, the principles, practices and values of health promotion include intersectoral collaboration, partnerships, advocacy, community capacity, empowerment and action research [15]. Thus, engaging many different perspectives and employing participatory methods is fundamental. Engaging with diverse perspectives is also integral to taking a complex systems approach. For example, Jackson [16] describes complex systems as having a large number of subsystems that interact and outcomes that cannot be predetermined, therefore, ‘sufficient accommodation between different and sometimes conflicting world views’ (p. 22) is necessary for applied systems change. Participatory methods are flourishing in system change efforts and include collective decision-making [7], co-production, co-creation and/or co-design [4, 17, 18]. Taking an explicit complex systems approach to health promotion and NCD prevention can build upon the traditions of both fields.

While complex systems approaches can facilitate a more holistic understanding, the optimal methods to study specific areas of health promotion, such as population physical activity (PA), are less clear. Physical inactivity is a leading risk factor for premature death and it ‘is estimated that between four and five million deaths per year could be averted if the global population was more active’ [19]. PA can be seen as an emergent property or the result of interactions inherent in socio-ecological systems (e.g. increased individual active commuting behaviour may result from a combination of strengthened community action for enhanced public transportation, local municipal laws and regulations regarding traffic calming, and regional or provincial funding for designated cycling lanes and paths). Taking this view led us to research what complex systems methods are used in PA research and which align best with a holistic or whole system approach.

**Fig. 1:**
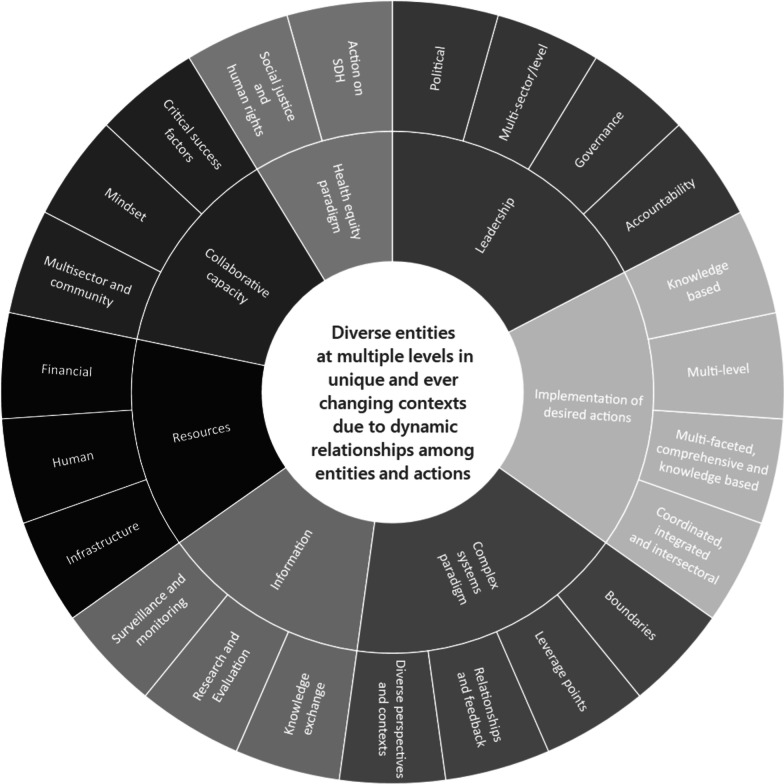
Attributes of effective systems for health promotion and NCD prevention [20]

A model developed by Baugh Littlejohns and Wilson [20] offers one way to examine complex systems for PA promotion. The Attributes Model consists of seven characteristics or attributes of effective systems that include information, leadership, implementation of desired actions, collaborative capacity, resources, health equity paradigm and complex systems thinking, as well as 23 associated dimensions (Fig. 1). Each attribute and associated dimension is described through examples in the results section. This model offers a way to study *complex systems* as opposed to the study of *complex problems*. The model directs attention to the interactions and interdependence among attributes. It is within these dynamic relationships where leverage points can be revealed and policy and practice options can be identified to strengthen systems for PA promotion (e.g. where action could produce significant impact to the whole system).

Our aim was to examine the types of complex systems methods used in current PA research and explore alignment with a whole of systems approach as described by the Attributes Model [20]. A recently published literature review on systems approaches to PA promotion [21] complements and validates our aim, in that, there is a need to ‘address systems-level enablers that arguably include governance and leadership, legislation and regulation, multisectoral partnerships, workforce capabilities, advocacy, information systems, system surveillance and financing mechanisms’ (p. 12).

## Methods

### Scoping review

The scoping review method was adopted as it can facilitate examination of how research is conducted on a topic and identify types of evidence in a given field [22]. Scoping reviews can be characterized as having (a) a priori review protocol, (b) an explicit, transparent search strategy and (c) a standardized data extraction process [22]. These characteristics are described below. A Preferred Reporting Items for Systematic Reviews-Scoping Reviews (PRISMA-SCR) checklist is included as Additional file 1: File S1.

### Search strategy

The Ovid Medline and Web of Science databases were selected for the search strategy, as they were considered to be comprehensive for our topic. A search strategy was developed and can be found in Additional file 1: File S2. The search was run in November 2021 and alerts were set up for each database to gain further articles published up to mid January 2022.

**Fig. 2:**
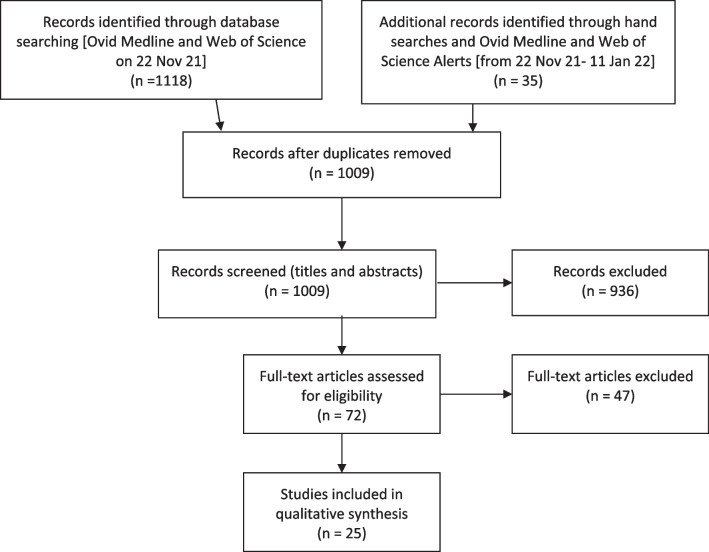
PRISMA flow diagram

We recorded 1153 articles through the search strategy (Fig. 2). Following removal of duplicates, a total of 1009 articles remained. Of the 1009 articles, titles and abstracts were reviewed by the first author (L.B.L.) based upon three inclusion criteria: (1) Explicitly used a complex systems research method (i.e. not including review articles), (2) investigated PA as the sole focus of the research or addressed PA in a substantive manner in an integrated study (e.g. obesity prevention) and (3) peer reviewed journal article published in English from January 2010 to January 2022. This timeframe was selected to include most recent literature and capture the latest innovations in using complex systems research methods.

From the review of titles and abstracts, 936 articles were excluded leaving 72 for full text review. Two authors (L.B.L. and E.N.) reviewed these articles and 47 were further excluded using the inclusion criteria above. This left 25 articles to be included in the final review (Fig. 2).

### Data extraction and analysis

Duplicate independent data extraction and validation was conducted by two authors (LBL and EN). Data extraction included copying and saving verbatim statements to an Excel spreadsheet. Standardized data extraction included the following four categories: (1) complex systems research methods used, (2) research aims (i.e. study PA as a sole focus or part of an integrated study such as obesity prevention), (3) if participatory methods were used and (4) findings, discussion and conclusions regarding attributes of systems. Both LBL and EN read all articles and discussed individual articles to gain clarity, however, each was responsible for data extraction of approximately half of the 25 articles. LBL and EN cross-checked all data extraction. To enhance reliability of data extraction, four articles were sent to other authors (DR, GM, GF and DN) for review of research methods used and key findings. This process enabled discussion and agreement on data extracted. LBL and EN completed summaries of each of the above categories for each article and saved them to an Excel spreadsheet. All summaries were cross-checked for accuracy and clarity.

## Results

### Research methods

Three general categories of methods were described across the 25 articles included in the study: system mapping (*n* = 11), simulation modelling (*n* = 10) and network analysis (*n* = 4) (Table 1).

**Table 1:** Methods used, reasons for using complex systems methods, participatory methods and research aims

Research method	Subtype		First author	Main reasons for using research method	Participatory methods	Research aims
System mapping (*n* = 11)	Group model building (*n* = 5)	Causal loop diagram and behaviour over time graph	Brennan (2015)	Understand complex system (understand insights from multiple communities regarding behaviours of systems affecting health); identify causal relationships among variables	Yes	To report on an evaluation of a multi-community healthy eating and active living community-based initiative; integrated study
		Causal loop diagram	Guariguata (2021)	Understand complex problem; identify intervention points and causal pathways	Yes	To report on factors that influence population physical activity and identify potential areas for intervention; sole focus on PA
		Causal loop diagram and behaviour over time graph	Keane (2015)	Understand complex problem (identify perceived influences on active living and healthy eating and how those influences change over time); identify feedback loops	Yes	To report on the evaluation of a healthy eating and active living initiative in one community; integrated study
		Behaviour over time graph	Hoehner (2015)	Understand complex system (study system behaviour related to policies, environments, collaborations and social determinants, and compare trends among multiple communities); create a visual of how a variable changes over time	Yes	To report on perceived trends in system behaviour regarding past, current and future changes over time related to policy, systems and environment in terms of healthy eating, active living and childhood obesity; integrated study
		Causal loop diagram	Waterlander (2021)	Understand complex problem (increase understanding of the complexity of obesity-related behaviours); Vvsualize elements in a holistic system and their causal relationships	Yes	To study the complexity of obesity-related behaviours in youth (diet, physical activity, sedentary behaviour, sleep); integrated study
	Other mapping methods (*n* = 6)	Conceptual map (with policy audit)	Bellew (2020)	Understand complex problem (understand obesity and physical inactivity); identify the pathways to possible solutions	No (but group sense making after maps created)	To describe a national project in terms of a high-level conceptual systems map including physical activity influences, governance, translation, advocacy mechanisms and system intervention points for policies and programmes; sole focus on PA
		Causal pathways diagram (with surveying and multilevel modelling)	Carlson (2012)	Understand complex system (understand the relationships between the built environment and physical health); create causal pathways and feedback loops	No	To describe how the built environment affects behaviour and health by conceptualizing how physical and perceived barriers impact the relationship between destination walking and self-reported health status; integrated study
		Conceptual map	Cavill (2020)	Understand complex system (portray various interactions that constitute the system); promote cross-sectoral systems thinking; support future planning and implementation of actions	No (but group sense making after maps created)	To report on the value of system mapping in a city-wide population physical activity promotion programme; sole focus on PA
		Concept map	Holdsworth (2017)	Understand complex problem (visualize relationships between concepts such as clusters of factors for dietary and physical activity behaviour)	Yes	To report on factors that influence dietary and physical activity behaviour in ethnic populations; integrated study
		Mapping policy and practice	Murphy (2020)	Understand complex system (visualize nonlinearity in systems); ensure cross sectoral collaboration, coproduction, knowledge sharing, and defining roles; identify intervention design	Yes	To describe the process and results of a systems approach to enhance multisectoral communication and identify current good practices and future action for promoting population physical activity using the Global Action Plan on Physical Activity (GAPA) framework; sole focus on PA
		System map	Signal (2012)	Understand complex system; co-create illustrations of systems /control parameters; recommend interventions	Yes	To identify possible public policy interventions targeting food security and physical activity and illustrate systems, control parameters, and interventions; integrated study
Simulation modelling (*n* = 10)	Agent-based modelling (*n* = 5)		Almagor (2021)	Understand complex system (simulate interactions of actors with one another and with the environment); explore potential impact of interventions in several domains	No	To simulate the impact of various physical activity interventions (active travel, outdoor play, school physical education and their combination) on children’s daily activities in an urban environment; sole focus on PA
			Frerichs (2020)	Understand complex system (create prototype that emulates a system); develop models to influence decision making; pilot participatory approach to increase participants’ understanding of elements of an agent-based model	Yes	To describe participatory methods for creating a basic structure of a model regarding physical activity; sole focus on PA
			Garcia (2018)	Understand complex problem (study patterns of leisure time physical activity considering the interactions among individual psychological attributes and built and social environments; understand interrelations and impacts of factors at different levels); identify interventions	Yes	To simulate population patterns of leisure time physical activity among adults taking into consideration the interaction between individuals’ psychological attributes and the built and social environments in which they live; focused solely on PA
			Orr (2016)	Understand complex problem (study characteristics of the obesity system through multilevel analysis); identify policy options in terms of impact on body mass index of Black and White people	No	To simulate the impact of policies with respect to PA, neighbourhood food and educational environments on Black/White disparities in body mass index; integrated study
			Salvo (2021)	Understand complex system [identify sustainable development goals (SDG) that may benefit from PA strategies and the multiple sectors and systems at play]; simulate the effects of interventions or strategies on recreational- and transportation-based PA and six SDG-related outcomes	No	To simulate the impacts of physical activity promotion strategies on SDG-related outcomes across high-, middle- and low-income country city types, and provide recommendations for future research, policy and practice; sole focus on PA
	System dynamics modelling (*n* = 4)	With group model building and causal loop diagram	MacMillan (2014)	Understand complex problem (compare policies, incorporating feedback effects, nonlinear relationships and time delays between variables); involve policy, community and academic stakeholders in participatory modelling	Yes	To simulate the effects of five active transportation policy scenarios for commuter bicycling on injury, physical activity, fuel costs, air pollution and carbon emissions outcomes; integrated study
		With behaviour change over time graphs and prevalence/incidence diagrams	Powell (2017)	Understand complex problem; demonstrate how different models impact childhood obesity over time; identify policy options	No	To simulate the impact of various policies on the future prevalence of childhood obesity; integrated study
		With causal loop diagram	Soler (2016)	Understand complex problem; forecast the impact of various policies on outcomes (estimate premature deaths and medical and productivity costs)	No	To report on (1) short-term benefits of interventions targeted to decrease obesity by increasing physical activity, improving nutrition, decreasing tobacco use or decreasing exposure to second-hand smoke; and (2) long-term benefits in terms of health outcomes; integrated study
		With causal loop diagram	Yang (2019)	Understand complex problem (understand system structure and the dynamic interaction of multiple variables)	No	To simulate how multilevel factors (i.e. urban design, urban sprawl, economic development, crime) influence and impact active travel to school and health behaviours of children; integrated study
	Cross impact balance (*n* = 1)	With group modelling and causal loop diagram	Stankov (2021)	Understand complex problem (understanding of urban health issues); examine interactions among system elements and future scenarios	Yes	To study the strength and nature of relationships among factors that influence the transportation and food systems and identify future scenarios; integrated study
Network analysis (*n* = 4)	Social network analysis (*n* = 3)	With system inventory of actions	Blackford (2021)	Understand complex system (examine types of relationships within a network and increase understanding of network operations and the roles of key actors or organizations); identify strategies to strengthen networks; inventory or audit the physical activity, nutrition and obesity prevention initiatives taking place	No	To inventory current actions, examine networks and identify potential strategies for improving the obesity prevention system; integrated study
		With system inventory of actions	Jancey (2021)	Understand complex system; inform policy and practice to improve obesity prevention interventions through increased understanding of network relationships	No	To report on network relationships to inform policy and practice regarding (physical activity and nutrition) obesity prevention; integrated study
			Marks (2018)	Understand complex system (describe network structures as a measure of community capacity to implement and sustain interventions)	No	To report on the structure of professional obesity prevention networks as a measure of potential capacity to implement interventions; integrated study
	Comparative network analysis (*n* = 1)	Network analysis	McGlashan (2018)	Understand complex system (compare thematic clusters identified by expert-driven and community-created system maps as to their size and strength of causal relationships)	No	To compare the expert-driven foresight obesity system map with community-based causal loop diagrams to determine similarities and differences; integrated study

### System mapping

System mapping methods were most frequently used (*n* = 11); although there was considerable heterogeneity among subtypes (Table 1). Five of the articles used group model building (GMB) [23–27]. This distinctive method can be defined as a way ‘to capture and synthesize the different so-called mental models of each stakeholder regarding causal pathways at work in systems and specifically, to develop causal loop diagrams (CLD)’ [26] (p. 722). A CLD was described by Waterlander et al. as a tool to visualize and ‘explore the multiple, interacting feedback loops operating in a system of interest’ [27] (p. 2). Behaviour over time graphs are a tool used in conjunction with GMB, and Hoehner et al. stated that these are a way to create ‘a picture of how a variable changes as time progresses’ [25] (p. 46).

All five GMB articles reported using participatory methods. The main reasons for using GMB were to understand either a complex problem [24, 26, 27] or a complex system [23, 25], although the lines between the two were often unclear (and in all methods reviewed). Other reasons included identification and visualization of feedback and causal relationships [24, 26, 27], identification of interventions [26] and comparison of trends over time [25]. Articles that explicitly aimed to identify interventions described ‘actions’ with respect to policies, programmes, strategies, regulations and laws that influence PA behaviour [20]. We found only one of the five GMB articles focused solely on PA [26].

Six articles reported using other mapping methods [13, 28–32] (Table 1) which did not focus on creating a CLD. Bellew et al. [28] and Cavill et al. [30] used conceptual maps to describe relationships among factors that influence PA. Causal pathway diagramming was used by Carlson et al. [29] as a way to visualize how the built environment affects behaviour and health. Concept mapping was defined by Holdsworth et al. [31] as ‘gathering and analysing different types of data and integrating these with prior research and experience’ to visualize complex systems (p. 3). Murphy et al. [13] mapped current and desired future policy and practice to the WHO’s Global Action Plan for Physical Activity [33]. A system map was described by Signal et al. [32] as a way to understand system parameters that control or influence other elements.

The main reasons for using other mapping methods were to understand a complex problem [28, 31] or a complex system [13, 29, 30, 32] (Table 1). Other stated reasons included identification of interventions [13, 28, 30, 32], identification and visualization of feedback and causal relationships [13, 29, 31], and promotion of system thinking [30]. Of these six articles, three articles used participatory methods [13, 31, 32]; however, it should be noted that Bellew et al. [28] and Cavill et al. [30] used forms of group sense making after system maps were created. Regarding research aims, half (*n* = 3) focused solely on PA [13, 28, 30].

### Simulation modelling

Simulation modelling was used in 10 of the 25 articles. Three subtypes were applied: five used agent-based modelling (ABM) [34–38], four used system dynamics modelling (SDM) [39–42] and one described a cross impact analysis [43] (Table 1). ABM can be defined as developing ‘computational models that simulate complex social systems by representing agents that interact with one another and with the environment in which they live according to predefined rules’ [34] (p. 2). ABM was used to simulate interactions among variables for validation and scaling up of the scenarios studied. There was little difference in terms of aims to either understand a complex system [34, 35, 37] or a complex problem [36, 38]. All articles, except Stankov et al. [43], explicitly indicated that identification of interventions was a key reason for using ABM. Two articles reported on participatory methods [35, 44] and all but Orr et al. [36] focused solely on PA.

SDM can be described as developing ‘a set of integral equations whose solutions are approximated to demonstrate dynamic system behaviour’ and solutions can enable ‘curves of trends over time in outcomes of interest to be explored and compared for future policy options’ [39] (p. 336). All four SDM articles were integrated studies and focused on understanding complex problems. One article used participatory methods [39].

The other simulation modelling method involved cross impact analysis, which Stankov et al. described as ‘a family of methods that can be used to afford insights into the possible future states of systems while accounting for mutual interactions between system factors’ [43] (p. 2). They studied a complex problem, used participatory methods, and the research was an integrated study.

### Network analysis

Network analysis was used in four of the 25 articles [45–48] (Table 1). All reported on social network analysis methods except for one [47]. Social network analysis can be described as the study of ‘the general structure of the network through lenses of average degree, density, diameter and reciprocity’ [48] (p. 3), whereas McGlashan et al. [47] used a comparative network analysis method which involved comparing and contrasting expert-driven and community-developed causal loop diagrams (as networks) to identify central variables. All four articles focused on understanding complex systems and examining network relationships. Network analysis was also used to identify interventions [45, 46, 48]. None of the five network analysis articles used participatory methods and none of the research focused solely on PA.

To summarize, articles reporting on system mapping methods appeared to align best with a whole system approach to PA promotion because they largely aimed to understand complex systems (7/11) [although the distinction between addressing complex systems and problems (e.g. topic) was often not clear cut], examined interactions and feedback among variables (6/11), and used participatory methods (10/11). Most of these articles focused solely on PA (as opposed to integrated studies) (6/11) and were less likely to concentrate on identifying interventions to influence PA behaviour (5/11). Simulation modelling methods were largely focused on examining complex problems (7/10) and identifying interventions (8/10). These methods did not generally focus solely on PA (1/10) or use participatory methods (4/10). However, with respect to the latter, some articles reported participatory methods such as in MacMillan et al. [39] where group model building was done as a precursor to modelling. Finally, while network analysis articles focused on examining complex systems (4/4) and identifying interventions (4/4), they did not focus solely on PA (0/4) nor use participatory methods (0/4).

### Attributes

All attributes were discussed across the 25 articles (Table 2). Attributes were either explicitly reported on in terms of findings (less so) or were part of the discussion and conclusion sections (more so). Examples include: *Implementation of Desired Actions* [23, 24], *Complex Systems Paradigm* [27, 31], *Leadership* [13, 26], *Information* [25, 30], *Collaborative Capacity* [28], *Resources* [29] and *Health Equity Paradigm* [32, 37]. We did not find this pattern in other methods. Brennan et al. [23] used group model building and discussed all attributes: *Complex System Paradigm* (e.g. identify causal relationships among variables), *Implementation of Desired Actions* (e.g. health behaviours, active living policies and environments), *Collaborative Capacity* (e.g. partnerships, community/civic engagement, social ties), *Resources* (knowledge and skill, financial/in kind resources), *Information* (research and evaluation), *Leadership* (political will, community leadership) and *Health Equity Paradigm* (e.g. social determinants of economy, employment, public transportation, targeted support to poor families, access to opportunities, neighbourhood associations). Articles that reported on simulation modelling methods appeared to align most with discussions of *Implementation of Desired Actions* and network analysis with *Collaborative Capacity*. The following provides examples of how attributes and associated dimensions were discussed.

**Table 2:** Key findings regarding physical activity promotion and examples of discussion of attributes

Research method	Subtype	First author	Key findings	Examples of discussion of attributes
System mapping (*n* = 11)	Group model building	Brennan (2015)	(1) The most common variables with respect to active living policies and environments were active transportation (e.g. access to public transportation, complete streets), recreation (e.g. access to parks, access to trails), community design and land use (e.g. urban sprawl, school siting), and motorized transportation (e.g. traffic safety, car dependence); (2) common variables regarding partnerships and community capacity were community organizing and advocacy (e.g. political will), youth and civic engagement, and community leadership; (3) common variables with respect to social determinants of health included harmful social conditions, beliefs, crime, poverty and segregation; (4) health behaviour variables included sedentary behaviours (e.g. driving, screen time)	Implementation of Desired Actions – multiLevel, comprehensive (e.g. health behaviours, active living policies and environments); Collaborative Capacity (e.g. partnerships, community/civic engagement, social ties); Resources (knowledge and skill, financial/in kind resources); Information (research and evaluation); Leadership (political will, community leadership); Health Equity Paradigm (e.g. social determinants of economy, employment, public transportation, targeted support to poor families, access to opportunities, neighbourhood associations) Information – Research and Evaluation × Complex Systems Thinking: Map systems to (a) increase understanding and communication of how actions are connected and how they can ‘synergistically impact’ systems, and (b) plan, implement and evaluate multifaceted actions to target policy, system structure and behaviour, and environmental variables that influence population physical activity
		Guariguata (2021)	(1) Cultural norms discourage physical activity [i.e. negativity towards sweating which influences active transport, associated with low socio-economic (SE) status]; (2) cultural norms are stronger for women and they have less time for physical activity; (3) ample space for physical activity but not always well maintained, safe or accessible to the public; (4) humid tropical climate is not conducive to physical activity and supports car use; (4) nested feedback loops illustrate needed multisectoral, multilevel and multipronged actions	(1) Complex systems thinking – diverse perspectives, relationships and feedback: diverse perspectives are important for ‘knowledge of different aspects of a system’, ‘breadth of experience’, ‘local knowledge’, developing ‘a broader, more systemic view’ and engaging those ‘empowered to enact or influence policies or interventions’; disrupt reinforcing feedback loops (e.g. with respect to cultural norms, physical activity and motor vehicle use); (2) implementation of desired actions – comprehensive: implement (a) community events to enhance supportive environments (e.g. especially for women, from small community-based initiatives in public spaces to the development of physical education in schools), (b) country-wide mass communication campaigns and (c) actions to reduce street crime and car use (e.g. financial incentives, public transit); (3) resources – infrastructure: integrate spaces for physical activity into communities (not just for tourists); (4) leadership – multisector/level × collaborative capacity – multisector and community × implementation of desired actions: policy leadership is needed on a regional level to support multisectoral collaboration and implementation of desired actions
		Keane (2015)	(1) Excessive time on the school bus was linked to inactivity; (2) after-school buses allowed for extracurricular physical activities and participation in health promotion programmes, which might lead to improved academic performance; (3) support for a curriculum that blends academics and physical activity; (4) more safe places to be physically active was linked to increased activity levels	Implementation of desired actions – comprehensive, coordinated, knowledge-based: implement school bus scheduling, curriculum and safe environments
		Hoehner (2015)	(1) Positive, increasing and reinforcing trends (graphs with increasing trend lines) were found to be most prevalent with respect to environments for active living (i.e. access to parks, park maintenance, bike infrastructure, bike share, urban sprawl, blight, sidewalks, crosswalks); (2) trends with respect to active living behaviour (i.e. children’s physical activity, walking to school, TV time, play outside) were found to be most prevalent as negative, decreasing and balancing (graphs with decreasing trend lines)	Information – research and evaluation, knowledge exchange × implementation of desired actions: behaviour-over-time graphs ‘serve as useful tools for describing the interrelated sources and consequences of complex behaviors, such as obesity, for the purposes of informing decisions and policies’ (p.53)
		Waterlander (2021)	(1) The social norms towards walking/cycling affects perceived safety and this influences walking/cycling behaviour, which in turn affects social norms; (2) system change with respect to macroeconomics, social welfare, technology and urban systems is needed rather than a focus on interventions targeting individual behaviour change	(1) Implementation of desired actions × complex systems thinking – boundaries, relationships and feedback: set boundaries guided by determinants that we can change, that are relevant to our population and are related to the target behaviours, at the level of family, school, neighbourhood, healthcare and city; implement actions focused on (a) changing the social norms among adolescents with respect to ‘normative physical activity’ through drawing attention to the neoliberal paradigm present in social media, marketing and policy options, (b) disrupt reinforcing feedback loops that characterize macro-level influences such as economic and urban systems, and (c) disrupt the reinforcing feedback loop of negative social norms towards walking/cycling behaviour and perceived safety
	Other mapping	Bellew (2020)	(1) Influences on physical activity included individual physiology, individual psychology, personal demographic status, social environment and norms, physical activity infrastructure and built environment, governance, knowledge translation, advocacy mechanisms, and system intervention points for policies and programmes (i.e. settings and sectors)	(1) Leadership – governance: governance structures and advocacy mechanisms are critical; (2) information – knowledge exchange: enhance knowledge translation; (3) collaborative capacity – multisector and community × implementation of desired actions – multilevel: consider settings and sectors as intervention points for policies and programmes
		Carlson (2012)	(1) The strongest associations were with respect to destination walking, sidewalks and connectivity (e.g. less places to walk was associated with less walking – to fewer locations, and less frequently; (2) a relationship was found with increased local walking and support for improving the local walking infrastructure; (3) increased walking in an area may increase the perception that the area is walkable	(1) Resources – infrastructure: built environment, sidewalks and connectivity; (2) implementation of desired actions – knowledge-based: community perceptions are important; (3) complex systems thinking – relationships and feedback: ‘Destination walking, health, and the built environment are likely related in a nonlinear, complex way’ (p. 279); Less places to walk negatively influences walking behaviour which influences places to walk
		Cavill (2020)	(1) Three specific domains of physical activity were identified: walking for transport, cycling for transport, and sport and active recreation; (2) broadening the range of data is necessary (e.g. quality of parks and green spaces, social norms for physical activity); (3) most actions to promote physical activity were focused at the interpersonal level	(1) Information – surveillance and monitoring, knowledge exchange: enhance data collection in areas such as social norms that support physical activity and city-level data (e.g. traffic, walkability, air quality, cycling infrastructure); (2) implementation of desired actions – comprehensive: implement a range of actions that address the influence of built and natural environments, social norms and interpersonal factors on physical activity
		Holdsworth (2017)	Eight clusters were identified with respect to factors that influence physical activity behaviours of ethnic minority populations and these are (in order of overall ranking as to priorities for research and interventions): (1) psychosocial, (2) institutional environment, (3) political environment, (4) social and cultural environment, (5) physical environment and opportunity, (6) social and material resources, (7) health and health communication, and (8) migration context	(1) Complex systems thinking – relationships and feedback: illustrate systems in terms of interrelated factors as a precursor to developing interventions, (2) implementation of desired actions – comprehensive, coordinated, multilevel, knowledge-based: consider (a) adapt interventions for the whole population to be diversity sensitive or equally effective for all citizens regardless of their cultural, religious or ethnic background, and/or (b) develop ‘migrant-specific’ interventions by culturally adapting services and interventions to minority ethnic groups
		Murphy (2020)	(1) Actions for greater impact may well lie with an ‘active system’ approach including (a) enhanced support and the renewal of policies and governance structures; (b) increased support for collaboration across sectors; (c) funding or dedicated budgets for advocacy, interdisciplinary policy actions and research development; (2) an active systems approach is closely linked to the creation of ‘active environments’ (e.g. additional funding and organizational support for strengthening policy, regulatory and design guidelines for PA engagement in and around public buildings and public places, and the improvement of walking and cycling infrastructure); (3) improvements to walking and cycling network infrastructure were identified as important actions for impact	(1) Leadership – governance × collaborative capacity – multisectoral and community: enhance governance structures to adopt an active systems approach and increase support for multisectoral collaboration; (2) implementation of desired actions – knowledge-based × resources – financial, infrastructure: gain financial resources to implement improvements to walking and biking infrastructure
		Signal (2012)	(1) Improve urban design (e.g. open space, connectivity) and (2) develop culturally specific physical activity programmes using cultural practices	(1) Implementation of desired actions – coordinated and comprehensive; implement coordinated and comprehensive actions across multiple levels of governance; (2) complex systems thinking – leverage points: prioritize interventions that impact on highly linked elements of systems; (3) leadership – political: advocate for strong government leadership; (4) health equity: ensure there is an explicit equity focus; (5) information – research and evaluation: use mixed methods to provide rich data; (6) collaborative capacity – multisectoral and community: ensure active participation of communities and policy makers
Simulation modelling	Agent-based modelling	Almagor (2021)	(1) Outdoor events in neighbourhoods can enhance the engagement of children in physical activity; (2) encouraging children to be active in diverse groups will likely have a positive effect on the least active; (3) the most important characteristic in influencing PA levels was found to be the agent’s tendency to be active; (4) the second most important factor for PA was the walking time of the agent; (5) ‘Outdoor play in the neighbourhood’ scenario demonstrated that increasing the frequency of outdoor play contributed to population PA beyond the direct engagement in the activity itself; (6) those with a higher SE position were more likely to take part in physical activity and formal sport activities	(1) Resources – infrastructure: create infrastructure that supports active travel along routes frequently used by children, (e.g. wide sidewalks, controlled road crossings, zones of reduced traffic, controlled speed and streets closed for vehicle traffic); (2) implementaton of desired actions – coordinated: implement ‘catalyst’ events (e.g. community get-togethers, street closure events) that could attract children and potentially trigger a positive feedback loop re: more outdoor play; (3) health equity: target lower SE subpopulations and create supportive environments for physical activity
		Frerichs (2020)	(1) There are time periods with more (after school) and less (during school) variation in daily activity, (2) key locations (i.e. school, home) most relevant to sedentary and physical activity, and (3) social interactions that were likely to influence physical activity choices	(1) Information – knowledge exchange × implementation of desired actions – implement participatory approaches to co-develop visual representations of models to deepen understanding of the influence of social interactions and spatial locations of physical activity and to support the identification of desired actions
		Garcia (2018)	(1) ‘Three elements and mechanisms exhibited stronger influence on time trends of people practicing LTPA [leisure-time physical activity] and levels of intention: the influence of the person’s behavior in the previous week over his current intention, size of the person’s perception radius, and proportion of LTPA sites in the model’ (p. 9); (2) Three other elements and mechanisms had lower effect: ‘proximal network’s and perceived community’s behaviors influence on the person’s intention, and mean quality score of LTPA site’ (p. 9)	(1) complex systems thinking – relationships and feedback: (a) the stronger the social influence, the higher the proportion of people with low intention to practice leisure-time physical activity (LTPA), (b) psychological attributes were found to be ‘the strongest proximal determinants of LTPA, however, this relationship is dynamically moderated by the built environment and influenced by both the social environment and the behavior itself’ (p. 9)
		Orr (2016)	(1) Physical activity infrastructure policy was found to have the greatest impact on the reduction if disparities [using a body mass index (BMI) disparity index]	(1) Implementation of desired actions × resources – infrastructure × health equity: enhance physical activity infrastructure and reduce disparities using a seven point neighbourhood environment index
		Salvo (2021)	(1) Comprehensive physical activity promotion strategies (i.e. at-scale strategies centered on transport systems that prioritize walking, cycling and transit; activity-promoting urban design; whole-school approaches; physical activity promotion in primary care; mass media campaigns and sports-for-all programmes) could provide benefits for LIC, MIC and sprawling HIC city types both in terms of physical activity participation and SDG improvements; (2) cities in Low-and Middle-Income Countries (LMICs) may accrue greater benefits from ‘scaled-up, synergistic physical activity promotion strategies than sprawling, car-centric city types in HIC’ (p. 1171)	(1) Implementation of desired actions – comprehensive, coordinated: implement (a) a multifaceted portfolio of actions, (b) ‘well-orchestrated’ actions and (c) ‘cross-sectoral’ actions; (2) collaborative capacity – multisectoral and community × complex system thinking – diverse perspectives: build collaboration among diverse sectors and perspectives (beyond health centricity); (3) complex system thinking – relationships and feedback × health equity: take action re: ‘Resolving socio-economic and gender-based inequalities could help improve population levels of physical activity. Conversely, physical activity promotion strategies have the potential to reduce inequalities’ (p. 1163)
	System dynamics modelling	MacMillan (2014)	(1) The greatest impact on active transportation can be viewed in terms of policies and practices to physically segregate arterial roads (with intersection treatments), to lower speed and to make local streets bicycle friendly	(1) Implementation of desired actions: ‘Although our findings suggest that Auckland’s existing plan to develop a regional cycle network would likely have benefits, the simulation modelling suggests that it would not reverse the predicted business-as-usual increased rate of cycling injury. In contrast, a gradual transformation of all roads using best practice arterial and local street interventions could make a major contribution to regional transport targets’(p. 342)
		Powell (2017)	(1) Daily physical education at school; (2) integration of moderate-to-vigorous physical activity into elementary school classrooms would have the largest projected impact on the prevalence of childhood obesity	(1) Implementation of desired actions – comprehensive: implement multifaceted actions in elementary school settings
		Soler (2016)	(1) Large investments and sustained community preventive interventions could yield cost savings many times greater than the original investment over 10–20 years and avert 14 000 premature deaths, (2) the greatest impact in obesity interventions were to increase physical activity in schools and child care facilities and promote physical activity in communities	(1) Resources – financial: provide adequate financial resources, (2) implementation of desired actions – multifaceted, intersectoral: sustain implementation of actions that target increasing physical activity in schools, child care facilities and communities
		Yang (2019)	(1) Economic development and urban sprawl are more influential than urban design and crime in terms of influence on active transportation to school (ATS); (2) there is a linear relationship between ATS and childhood overweight and obesity; (3) as economic development, urban sprawl, crime and poor urban design increase, ATS decreases	(1) Leadership – governance, accountability × implementation of desired actions: implement policies to (a) slowdown massive roadway investment, (b) expand and improve public transport, cycling, and walking facilities, and (c) restrict motor vehicle use in congested areas; (2) Complex Systems Thinking – relationships and feedback: disrupt the balancing feedback mechanisms that hold systems in status quo (i.e. as economic development, urban sprawl, crime and poor urban design increase, ATS decreases)
	Cross impact analysis	Stankov (2021)	(1) The importance of gaining political will for social change; (2) low car use and high street safety from crime, high public transportation subsidies and more free time were associated with future scenarios characterized by favourable health outcomes (including low chronic disease prevalence, high physical activity and low processed food consumption)	(1) Leadership – political: foster political leadership; (2) implementation of desired actions: implement low car use initiatives and public transportation subsidies; (3) information – research and evaluation, knowledge exchange: provide information from research on factors that influence transportation systems, physical activity and health outcomes
Network analysis	Social network analysis	Blackford (2021)	(1) 50% (*n* = 95) of initiatives targeted physical activity, 35% (*n* = 66) targeted nutrition and 15% (*n* = 28) targeted both nutrition and physical activity; (2) most objectives targeted behaviour change, knowledge, skills and awareness; (3) the least common objectives were changes to the built environment, advocacy and regulations; (4) information and knowledge sharing networks were the most densely connected, whereas the networks for sharing resources and partnering in planning were less dense; (5) funding, staffing, collaboration, policy and ‘political feasibility’ were ranked as key contributors to effective implementation	(1) Implementation of desired actions – comprehensive, coordinated, multilevel: implement multifaceted interventions (e.g. from individual behaviour change through to creating supportive environment and building healthy public policy); (2) collaborative capacity – mindset × resources – financial, human: facilitate joint funding and planning across multiple organizations and initiatives
		Jancey (2021)	(1) Of the 35 prevention actions identified, 14 targeted physical activity; (2) the actions were predominantly media strategies and resource development; (3) collaboration was lower than expected as each of the organizations identified awareness of only 6/15 other organizations implementing action; (4) ‘while both core and periphery groups frequently selected limited funding and staffing as a barrier to implementing prevention activities, only periphery organizations indicated “insufficient collaborations and partnerships” and “insufficient community connections” as barriers’ (p. 6)	(1) Collaborative capacity – multisectoral and community, critical success factors × leadership – governance × implementation of desired actions: strengthen governance structures for collaboration and shared planning; (2) complex systems thinking – relationships and feedback: alter feedback mechanisms (e.g. lack of communication results in lack of collaboration which feeds back to lack of communication)
		Marks (2018)	(1) Community leadership networks for obesity prevention which included population physical activity were found to be ‘sparse and disconnected’	(1) Collaborative capacity – multisectoral and community: actively build collaboration among diverse people and sectors; (2) leadership – governance: consider impact of decentralized or centralized governance structures
	Comparative network analysis	McGlashan (2018)	(1) Physical activity was a key variable in both the community and foresight causal loop diagrams; (2) the community map indicated the influence of upstream, proximal environmental factors such as local infrastructure and cost of exercise; (3) community maps are based upon local understanding of situations and needs and may therefore result in more locally relevant and feasible intervention strategies	(1) Information – research and evaluation × collaborative capacity – multisector and community: conduct participatory action research; (2) implementation of desired actions – knowledge-based: implement strategies based upon community identified actions; (3) complex systems thinking – relationships and feedback: use causal loop diagrams as at tool to increase understanding of community contexts

LIC: Low Income Countries, MIC: Middle Income Countries, HIC: High Income Countries

### Complex systems paradigm

*Relationships and feedback* are key dimensions to the *Complex Systems Paradigm* attribute (Fig. 1). All articles used complex systems methods to examine relationships among variables in some way, with many offering detailed discussion of feedback. Feedback refers to circular causality, where one variable feeds back onto another resulting in virtuous or vicious cycles [49]. Almost all articles that reported on system mapping methods explicitly explored feedback, while those using simulation modelling did so to a lesser extent (Table 2). One example of explicit discussion of feedback was that having less destinations or places to walk negatively influenced walking behaviour, which in turn, negatively influenced actions to increase places to walk [29]. Articles that reported on network analysis discussed relationships but not feedback mechanisms.

*Leverage points*, in this study, are the interactions and feedback among attributes that may be amplified or disrupted for positive system change. The description by Signal et al. of prioritizing areas ‘that impact on highly linked elements of systems’ [32] aligns with the concept of leverage points, although the term was not explicitly used (p. 92). Table 2 reports on examples of linked attributes and dimensions, and examples of interactions and feedback among attributes are discussed below.

*Boundaries* relate to how systems are defined or ‘what’s in’, ‘what’s out’ and ‘who decides’. Defining boundaries in essence creates the mental model of the system under study. There was a wide range in terms of the extent to which boundaries were discussed. For example, precise variables selected by researchers were used in simulation modelling and network analysis, whereas system mapping methods tended to be less explicit. However, Waterlander et al. [27] explained that system mapping and creating CLDs were very dependent upon the boundaries they set (i.e. academic expert perspectives regarding a programme in three lower SE neighbourhoods in Amsterdam). Overall, detailed boundary descriptions were generally not well articulated.

The importance of having *diverse perspectives* was evident in many articles. Almost half the articles (12/25) reported on participatory methods (Table 1) and many argued for intersectoral collaboration and empowerment. For example, Guariguata et al. [26] stated that diverse perspectives are important for incorporating ‘knowledge of different aspects of a system’, ‘breadth of experience’, ‘local knowledge’, ‘a broader, more systemic view’ and engaging those ‘empowered to enact or influence policies or interventions’ (p. 726).

### Implementation of desired actions

As noted above, ‘action’ is a term used to describe all types of policies, programmes, strategies, regulations and laws to influence PA. Articles were only included in the review if they substantively addressed PA promotion. Therefore, *Implementation of Desired Actions* was addressed to some extent in all articles, either directly related to a finding or as part of discussion and/or conclusions about implications for policy and practice. This was the case even though a little over half of the articles (13/25) explicitly aimed to identify interventions. Table 2 provides a summary of key findings regarding PA promotion and examples of explicit discussion of *Implementation of Desired Actions* (and other attributes and dimensions) and interactions among attributes.

Overall, key findings addressed a range of topics regarding PA promotion including built environment and urban design [13, 25, 26, 28, 29, 31, 32, 36, 38, 45, 47]; schools [23, 24, 35, 37, 40–42]; active transportation [23, 30, 37, 39, 42]; socio–ecological perspectives [27, 30, 31, 44, 45]; safety, injury and physical environments [24, 26, 27, 39, 43]; social environments [26, 28, 35]; children [25, 34, 42] and sport [34, 37]. See Additional file 1: File S3 for a detailed summary of these topics and findings. In terms of dimensions (Fig. 1), Salvo et al. [37] offered an example of *Comprehensive* action. They stated the need for multifaceted actions or at-scale strategies centred on transport systems that prioritize walking, cycling and transit; activity-promoting urban design; whole school approaches; physical activity promotion in primary care; mass media campaigns and sports-for-all programmes. Examples of *Coordinated* and *Multilevel* actions included (a) the need for coordination in school bus schedules, curriculum and safe environments for PA [24] and (b) actions implemented across multiple levels of governance [32]. With respect to the dimension of *Knowledge-based* actions, it can be argued that all articles were concerned to some extent with evidence-based interventions and it is notable that two articles highlighted the need for practice-based evidence or community-identified strategies to guide implementation [35, 47].

There were many examples where *Implementation of Desired Actions* was explicitly discussed in terms of interacting with other attributes such as *Information* [23, 25, 35], *Complex Systems Paradigm* [23, 27], *Collaborative Capacity* [28, 48], *Resources *[13, 36] and *Leadership* [42, 48]. Jancey et al. [48] linked *Implementation of Desired Actions* with *Collaborative Capacity* and *Leadership* (i.e. need to strengthen governance structures for collaboration and shared planning to take action). Brennan et al. [23] linked this with *Information* and *Complex Systems Paradigm* where they called for mapping systems to (a) increase understanding and communication of how actions are connected and how they can ‘synergistically impact’ systems and (b) plan, implement and evaluate multifaceted actions to target policy, system structure and behaviour, and environmental variables that influence population physical activity (Table 2).

### Collaborative capacity

There was little explicit discussion of the need for a *Mindset* that demonstrates a shared sense of collaborative value [50], nor was there discussion of *Critical Success Factors* such as ongoing efforts to build trust. However, most articles discussed the importance of effective *Multisectoral and community* collaboration (Table 2). Network analysis methods appeared to be particularly aligned with this attribute as they focus on relationships in networks, collaboratives or systems. For example, Marks et al. [46] reported that networks for obesity prevention were sparse and disconnected and Blackford et al. [45] found that knowledge sharing networks were the most densely connected, whereas networks for sharing resources and partnering in planning were less dense.

### Health equity paradigm

Reducing health inequities or taking a social justice approach through action on the determinants of health was identified in five of the 25 articles [23, 31, 32, 34, 37] (Table 2). Three articles reported on system mapping methods and discussed the need for an explicit focus on culturally specific practices and activities [32]; harmful social conditions, beliefs, crime, poverty and segregation [23]; and whole population approaches that are diversity sensitive or equally effective for all citizens as well as migrant-specific, culturally adapted actions targeted to minority ethnic groups [31]. ABM was used in the other two articles [36, 39]. Almagor et al. [34] discussed health equity in terms of targeting lower SE subpopulations and creating supportive environments for physical activity. Two attributes appeared to be linked in Salvo et al. [37] where they described a feedback mechanism between *Health Equity Paradigm* and *Complex Systems Paradigm*: ‘Resolving socioeconomic and gender-based inequalities could help improve population levels of physical activity. Conversely, physical activity promotion strategies have the potential to reduce inequalities’ (p. 1163).

### Resources

*Resources* were discussed in nine articles: five reported on system mapping methods [13, 23, 26, 29, 31], three on simulation modelling methods [34, 36, 41] and one on network analysis [45]. Most discussion focused on *Financial* resources. For example, Soler et al. [41] argued that large investments and sustained community preventive interventions could yield cost savings. Funding or dedicated budgets for advocacy, interdisciplinary policy actions and research development was described by Murphy et al. [13]. The need to facilitate joint funding and planning across multiple organizations and initiatives was stated by Blackford et al. [45]. Specific attention to *Human resources* was not stressed in any of the articles. *Infrastructure* was clearly described in the numerous articles that reported on active transportation, built environment, urban design, safety and the physical environment. Technological or communication infrastructure was not discussed in the articles. An example of *Resources* linked with *Implementation of Desired Actions* and *Health Equity Paradigm* was found in the Orr et al. [36] article. Here they concluded the need for enhanced physical activity infrastructure in conjunction with reducing disparities using a seven-point neighbourhood environment index.

### Leadership

Two articles discussed *Political* will and leadership [23, 43]. For example, Stankov et al. [43] reported on the importance of leadership to foster the political will to plan and implement low car use initiatives and public transportation subsidies. The dimension of *Multisector and community* and *Multilevel* leadership was highlighted in many articles. Guariguata et al. [26] described the need for policy leadership on a regional level in the Caribbean to build *Collaborative Capacity* for the *Implementation of Desired Actions*. Bellew et al. [28] identified the need for effective *Governance* structures and advocacy mechanisms and Murphy et al. [13] identified *Governance* as important to the adoption of an active systems approach. *Governance* was also discussed in terms of considering the impact of decentralized or centralized governance structures [51]. None of the articles spoke to the *Accountability* dimension of leadership.

### Information

*Surveillance and monitoring* was only addressed by Cavill et al. [30]. They cited the need to broaden the range of data reported regarding the quality of parks and green spaces, social norms for physical activity and walkability. Direct discussion of *Research and evaluation* was found in several articles [23, 25, 32, 43, 47]. For example, Signal et al. [32] described the need for mixed methods to provide rich data and McGlashan et al. [47] argued for participatory action research methods. Attention to *Knowledge exchange* was found in several articles [25, 28, 30, 35, 43]. For example, Bellew et al. [28] identified this as one element in a system map of influences on PA. Frerichs et al. [35] discussed *Knowledge exchange* and *Implementation of Desired Actions* in terms of deepening understanding of influences on PA to support action.

## Discussion

This scoping review aimed to study the complex systems methods used in current PA research and explore what methods appear to best align with a whole systems approach as reflected in the Attributes Model. There is a paucity of literature that describes the use of complex systems methods in PA research, and those that we reviewed rarely provided a comprehensive analysis of the whole system. Nonetheless, some methods appeared more aligned with the Attributes Model.

System mapping and network analysis methods appear to be most aligned with the aim to understand *complex systems* as opposed to *complex problems*. However, theoretical underpinnings to studying complex systems versus complex problems were often unclear and we suggest that future research be more explicit or better articulate theoretical underpinnings. Meadows’ [52, 53] theoretical and practical framework of ‘places to intervene in systems’ provides a foundation for studying systems change efforts to promote PA. For example, this framework could be a helpful addition to the Attributes Model as interactions among attributes or leverage points could be explored through Meadows’ *places*, such as positive and negative feedback loops, information flows, system goals and paradigms.

Most articles reported on integrated studies, where the research aims were not focused solely on PA as a topic area. We acknowledge the tight interdependence of multiple risk factors in NCD prevention and the clear links to a holistic systems view. However, the lack of specific focus on PA appears to be consistent with statements that call PA ‘the Cinderella risk factor’ because it is often viewed as part of obesity prevention and not studied for the breadth of benefits in its own right [54]. Implications from this point to the continued need for more studies that focus on PA promotion from a whole systems approach to build more knowledge and experience [55–58].

Participatory methods were more frequently adopted in system mapping methods and these unsurprisingly also emphasized inclusion of diverse perspectives*.* There were many different participatory processes used in system mapping (and in some simulation modelling) and implications for future research suggest comparing and contrasting these in terms of practical and methodological considerations. Furthermore, the concept of human learning systems [59] was not explicitly considered, despite it being inherent in participatory system mapping methods. Attention to the significance of learning is described by Bowen and O’Doherty [60]:

‘Creating a clear, visual map of a system promotes learning by depersonalizing our own mental models and giving us a way to examine alternatives. Thus, the process of mapping the basic plumbing of a system can be a powerful leverage point in and of itself that can open a “flow” of learning’ (no page).

The three research methods have different purposes or address different types of research questions, and have particular strengths. This points to how they can be used to complement one another in a whole system approach. For example, we suggest starting with a focus on a complex system and using the Attributes Model as a platform for mapping. Other methods such as simulation modelling could be used to examine what should be implemented to address the complex problem (*Implementation of Desired Actions*) and/or network analysis to study characteristics of network relationships and how these findings relate to *Collaborative Capacity*. Power et al. [61] provide an example of the latter.

Finally, we found that system mapping methods were strongest in terms of the potential to fully examine the scope of attributes and the depth of their associated attributes (Fig. 1). The findings of this study demonstrate the value of the Attributes Model as a promising whole system approach. We note that a number of associated dimensions of attributes were not discussed, for example, accountability as a dimension of leadership was not addressed. This points to the potential of applying the the Attributes Model in its entirety to address these gaps. We plan to apply these findings to a project to strengthen systems for PA in British Columbia, Canada and believe they could be useful to other jurisdictional projects around the world.

### Limitations

We found 25 articles that met our inclusion criteria, and these provided a broad overview of complex systems methods explicitly used in PA research and the extent to which the research aligns with attributes of a whole of system approach. Two limitations are that the search strategy did not include grey literature nor articles not in English. These factors may have limited our findings. Furthermore, some articles were excluded because they did not explicitly use a robust complex systems research method. For example, they only discussed systems theory [55, 56] or used systems concepts in data analysis [62]. These types of articles may contain new insights for taking a whole systems approach.

Two databases were searched and although this might be seem to be a limitation, they were considered appropriate, particularly as the intent of this scoping review was ‘to acquire a broad sense of the state of the science rather than an exhaustive list of all articles published’ [37]. Finally, identifying and reporting on leverage points (i.e. interactions among attributes) was based solely upon our interpretation of findings, discussion and conclusions in the articles. This was clearly exploratory in nature and authors could well place different emphasis in their interpretations.

## Conclusions

Complex systems methods have the potential to enhance NCD prevention and PA promotion and whole systems approaches are thought to hold promise to enhance the integration of socio–ecological models. We found that system mapping methods were most aligned with this approach (as articulated in the Attributes Model). These types of methods were also most aligned with the importance of engaging diverse perspectives through participatory processes and identifying places to intervene in systems. Implications for future whole systems approaches to PA research and practice include the application of the Attributes Model in conjunction with system mapping methods (i.e. using all the attributes and associated dimensions as variables for mapping). Additionally, simulation modelling and network analysis methods were found to be complementary, and implications include using these methods when system mapping methods reveal further research questions. For example, research questions that focus on what actions (i.e. policies, programmes, strategies, regulations and laws to influence PA) should be implemented or how densely are relationships connected in systems.

In conclusion, there appears to be limited research that reports on a comprehensive whole of systems approach to PA promotion. This is an important finding given the growing interest and promise of these methods. A key implication to advance research, policy and practice is to undertake a comprehensive approach. This would include an iterative process to describe systems in terms of attributes and associated dimensions, assess relationships and feedback mechanisms among attributes to identify key leverage points, and strengthen systems by making systemic changes in priority areas and evaluating impacts. Thus, conducting research through comprehensive whole systems approaches will require embedding researchers with policy makers and practitioners to establish human learning systems. We believe this scoping review offers important information and practical ways to take a comprehensive whole system approach to PA promotion.

## Supplementary Information

**Additional file 1: File S1.** Scoping Reviews (PRISMA-ScR) Checklist. **File S2.** Search strategy. **File S3.** Detailed summary of topics and key findings.

## Data Availability

All data generated or analysed during this study are included in this published article and its additional files.

## Abbreviations

ABM: Agent-based modelling
CLD: Causal loop diagram
GMB: Group model building
NCD: Noncommunicable disease
PA: Population physical activity
SDM: System dynamics modelling
