# Supplementary material for: A scoping review of complex systems methods used in population physical activity research: do they align with attributes of a whole system approach?
Source: Health Res Policy Syst. 2023 Mar 2;21:18. doi: 10.1186/s12961-023-00961-3 (PMC9979563; doi:10.1186/s12961-023-00961-3)
Supplement: Supplementary file 1 — Additional file 1: File S1. Scoping Reviews (PRISMA-ScR) Checklist. File S2. Search strategy. File S3. Detailed summary of topics and key findings. [file 12961_2023_961_MOESM1_ESM.docx]

ADDITIONAL FILE 1

## File S1. Scoping Reviews (PRISMA-ScR) Checklist

Preferred Reporting Items for Systematic reviews and Meta-Analyses extension for Scoping Reviews (PRISMA-ScR) Checklist

*From:* Tricco AC, Lillie E, Zarin W, O'Brien KK, Colquhoun H, Levac D, et al. PRISMA Extension for Scoping Reviews (PRISMAScR): Checklist and Explanation. Ann Intern Med. 2018;169:467–473. [doi: 10.7326/M18-0850](http://annals.org/aim/fullarticle/2700389/prisma-extension-scoping-reviews-prisma-scr-checklist-explanation)

| **SECTION** | **ITEM** | **PRISMA-ScR CHECKLIST ITEM** | **REPORTED ON PAGE #** |
| --- | --- | --- | --- |
| **TITLE** | | | |
| Title | 1 | Identify the report as a scoping review. | A scoping review was chosen as a method as it is designed to examine how research is conducted on a topic and to identify types of evidence in a given field. Scoping reviews can be characterized as having a) a priori review protocol, b) an explicit, transparent search strategy, and c) a standardized data extraction process. These characteristics are described below. The intent of a scoping review is “to acquire a broad sense of the state of the science rather than an exhaustive list of all articles published”. |
| **ABSTRACT** | | | |
| Structured summary | 2 | Provide a structured summary that includes (as applicable): background, objectives, eligibility criteria, sources of evidence, charting methods, results, and conclusions that relate to the review questions and objectives. | Introduction  Complex systems methods are increasingly being used in health promotion and non-communicable disease prevention research, policy and practice. Questions emerge as to the best ways to take a complex systems approach, specifically with respect to population physical activity (PA). Using an Attributes Model is one way to understand complex systems. We aimed to examine the types of complex systems methods used in current PA research and what methods align with a whole of system approach such as in the Attribute Model.  Methods  A scoping review was conducted and two databases were searched. Twenty five articles were selected and data analysis was based upon four categories: the complex systems research methods used, research aims, if participatory methods were used, and evidence of discussion regarding attributes of systems.  Results There were three groups of methods used: system mapping, simulation modeling, and network analysis. System mapping methods appeared to align best with a whole of system approach to PA promotion because they largely aimed to understand complex systems, examine interactions and feedback among variables, and used participatory methods. Most of these articles focused on PA (as opposed to integrated studies). Simulation modeling methods were largely focused on examining complex problems and identifying interventions. These methods did not generally focus on PA or use participatory methods. While network analysis articles focused on examining complex systems and identifying interventions, they did not focus on PA nor use participatory methods.  All attributes were discussed in some way in the articles. Attributes were either explicitly reported on in terms of findings or were part of the discussion and conclusion sections. System mapping methods appear to be well aligned with a whole of system approach because these methods addressed all attributes in some way. We did not find this pattern with other methods.  Conclusions  Future research should apply the Attributes Model in conjunction with system mapping methods. Simulation modeling and network analysis methods were seen as complementary and could be used when system mapping methods identify priorities for further investigation (e.g., what interventions to implement or how densely connected are relationships in systems. |
| **INTRODUCTION** | | | |
| Rationale | 3 | Describe the rationale for the review in the context of what is already known. Explain why the review questions/objectives lend themselves to a scoping review approach. | Complex systems methods are increasingly being used in public health, health promotion and non-communicable disease (NCD) prevention research, policy and practice. These methods answer the call to shift to incorporate holistic systems views and away from reductionist and linear cause effect approaches. Holistic or whole of systems approaches can be described as focusing on “people, processes, activities, settings and structures – and the dynamic relationships between them” (p.2). Calenbuhr explains that taking a whole of systems approach means phenomena such as emergent properties, evolutionary system change and collective decision making are central and a shift from the study of parts to the whole system is necessary. There are hundreds complex systems methods and approaches developed since at least the 1940’s and new innovations are emerging rapidly.  The question emerges as to what are the best ways to take a complex systems approach and in our case, to population physical activity (PA) research, policy and practice in British Columbia (BC); |
| Objectives | 4 | Provide an explicit statement of the questions and objectives being addressed with reference to their key elements (e.g., population or participants, concepts, and context) or other relevant key elements used to conceptualize the review questions and/or objectives. | Standardized data extraction included the following four categories: 1) complex systems research methods used, 2) research aims, 3) if participatory methods were used, and 4) findings, discussions and conclusions regarding attributes of systems. This standardized data extraction process was reviewed and approved by all authors**.** |
| **METHODS** | | | |
| Protocol and registration | 5 | Indicate whether a review protocol exists; state if and where it can be accessed (e.g., a Web address); and if available, provide registration information, including the registration number. | Duplicate independent data extraction and validation was conducted by two authors (LBL and EN). Data extraction included copying and saving verbatim statements to an Excel spreadsheet. Standardized data extraction included the following four categories: 1) complex systems research methods used, 2) research aims, 3) if participatory methods were used, and 4) findings, discussions and conclusions regarding attributes of systems. This standardized data extraction process was reviewed and approved by all authors**.** Both LBL and EN read all articles and discussed individual articles to gain clarity, however, each was responsible for data extraction of approximately half of the 25 articles. LBL and EN cross checked all data extraction. In order to enhance reliability of data extraction, four articles were sent to other authors (DR, GM, GF, and DN) for review of research methods used and key findings. This process enabled discussion and agreement on data extracted. LBL and EN completed summaries of each of the above categories for each article and saved to an Excel spreadsheet. All summaries were cross checked for accuracy and clarity |
| Eligibility criteria | 6 | Specify characteristics of the sources of evidence used as eligibility criteria (e.g., years considered, language, and publication status), and provide a rationale. | Of the 1009 articles, titles and abstracts were reviewed by the first author (LBL) based upon three inclusion criteria: 1) Explicitly used a complex systems research method; 2) Investigated PA as the sole focus of the research or addressed PA in a substantive manner in an integrated study (e.g., obesity prevention), and 3) Peer reviewed journal article published in English from 1 January 2010 to 11 January 2022. This timeframe was selected to include most recent literature and capture the latest innovations in using complex systems research methods. |
| Information sources* | 7 | Describe all information sources in the search (e.g., databases with dates of coverage and contact with authors to identify additional sources), as well as the date the most recent search was executed. | The Ovid Medline and Web of Science databases were selected for the search strategy based upon the recommendation from a university librarian. These databases were considered to be broad and comprehensive for our topic. A search strategy was developed and can be found in Additional File 2. The search was run in November 2021 and alerts were set up for each database to gain further articles published up to mid January 2022. Hand search of reference list and review of other articles found through author’s networks was also completed. |
| Search | 8 | Present the full electronic search strategy for at least 1 database, including any limits used, such that it could be repeated. | Ovid MEDLINE(R) and Epub Ahead of Print, In-Process, In-Data-Review & Other Non-Indexed Citations, Daily and Versions(R) and Web of Science databases were searched using the follow terms:  complex systems.mp. system* dynamics.mp. 1 or 2 Exercise/ Physical Fitness/ (physical activity or sedentary or physical fitness).mp. (complex adj2 systems).mp. 4 or 5 or 6 2 or 7 8 and 9 (systems science or systems thinking or systems practice).mp. 2 or 7 or 11 8 and 12 limit 13 to (english language and yr="2010 -Current") health promotion/ or healthy people programs/ health promotion.mp. chronic disease prevention.mp. 15 or 16 or 17 8 or 18 12 and 19 limit 20 to (english language and yr="2010 -Current") |
| Selection of sources of evidence† | 9 | State the process for selecting sources of evidence (i.e., screening and eligibility) included in the scoping review. | We recorded 1153 articles through the search strategy (Figure 2). Following removal of duplicates, a total of 1009 articles remained. Of the 1009 articles, titles and abstracts were reviewed by the first author (LBL) based upon three inclusion criteria: 1) Explicitly used a complex systems research method; 2) Investigated PA as the sole focus of the research or addressed PA in a substantive manner in an integrated study (e.g., obesity prevention), and 3) Peer reviewed journal article published in English from 1 January 2010 to 11 January 2022. This timeframe was selected to include most recent literature and capture the latest innovations in using complex systems research methods.  From the review of titles and abstracts, 936 articles were excluded leaving 72 for full text review. Two authors (LBL and EN) reviewed these articles and 47 were further excluded using the inclusion criteria above. This left 25 articles to be included in the study (Figure 2). |
| Data charting process‡ | 10 | Describe the methods of charting data from the included sources of evidence (e.g., calibrated forms or forms that have been tested by the team before their use, and whether data charting was done independently or in duplicate) and any processes for obtaining and confirming data from investigators. | Duplicate independent data extraction and validation was conducted by two authors (LBL and EN). Data extraction included copying and saving verbatim statements to an Excel spreadsheet. Standardized data extraction included the following four categories: 1) complex systems research methods used, 2) research aims, 3) if participatory methods were used, and 4) findings, discussions and conclusions regarding attributes of systems. This standardized data extraction process was reviewed and approved by all authors**.** Both LBL and EN read all articles and discussed individual articles to gain clarity, however, each was responsible for data extraction of approximately half of the 25 articles. LBL and EN cross checked all data extraction. In order to enhance reliability of data extraction, four articles were sent to other authors (DR, GM, GF, and DN) for review of research methods used and key findings. This process enabled discussion and agreement on data extracted. LBL and EN completed summaries of each of the above categories for each article and saved to an Excel spreadsheet. All summaries were cross checked for accuracy and clarity. |
| Data items | 11 | List and define all variables for which data were sought and any assumptions and simplifications made. | Standardized data extraction included the following four categories: 1) complex systems research methods used, 2) research aims, 3) if participatory methods were used, and 4) findings, discussions and conclusions regarding attributes of systems. This standardized data extraction process was reviewed and approved by all authors**.** |
| Critical appraisal of individual sources of evidence§ | 12 | If done, provide a rationale for conducting a critical appraisal of included sources of evidence; describe the methods used and how this information was used in any data synthesis (if appropriate). | n/a |
| Synthesis of results | 13 | Describe the methods of handling and summarizing the data that were charted. | Standardized data extraction included the following four categories: 1) complex systems research methods used, 2) research aims, 3) if participatory methods were used, and 4) findings, discussions and conclusions regarding attributes of systems. This standardized data extraction process was reviewed and approved by all authors**.** Both LBL and EN read all articles and discussed individual articles to gain clarity, however, each was responsible for data extraction of approximately half of the 25 articles. LBL and EN cross checked all data extraction. In order to enhance reliability of data extraction, four articles were sent to other authors (DR, GM, GF, and DN) for review of research methods used and key findings. This process enabled discussion and agreement on data extracted. LBL and EN completed summaries of each of the above categories for each article and saved to an Excel spreadsheet. All summaries were cross checked for accuracy and clarity. |
| **RESULTS** | | | |
| Selection of sources of evidence | 14 | Give numbers of sources of evidence screened, assessed for eligibility, and included in the review, with reasons for exclusions at each stage, ideally using a flow diagram. | 25 |
| Characteristics of sources of evidence | 15 | For each source of evidence, present characteristics for which data were charted and provide the citations. | 1) complex systems research methods used, 2) research aims, 3) if participatory methods were used, and 4) findings, discussions and conclusions regarding attributes of systems. |
| Critical appraisal within sources of evidence | 16 | If done, present data on critical appraisal of included sources of evidence (see item 12). | n/a |
| Results of individual sources of evidence | 17 | For each included source of evidence, present the relevant data that were charted that relate to the review questions and objectives. | Spreadsheets available upon request |
| Synthesis of results | 18 | Summarize and/or present the charting results as they relate to the review questions and objectives. | See Tables 1 and 2 in the manuscript |
| **DISCUSSION** | | | |
| Summary of evidence | 19 | Summarize the main results (including an overview of concepts, themes, and types of evidence available), link to the review questions and objectives, and consider the relevance to key groups. | To summarize, articles reporting on system mapping methods appeared to align best with a whole of system approach to PA promotion because they largely aimed to understand complex systems (7/11) (although the distinction with addressing complex problems was often not clear cut), examine interactions and feedback among variables (6/11), and used participatory methods (10/11). Most of these articles focused on PA (as opposed to integrated studies) (6/11) and were less likely to explicitly aim to identify interventions to influence PA behavior (5/11). Simulation modeling methods were largely focused on examining complex problems (7/10) and identifying interventions (8/10). These methods did not generally focus on PA (1/10) or use participatory methods (4/10). However, with respect to the latter some articles reported participation such as in MacMillan et al (1) where group model building was done as a precursor to modelling. Finally, while network analysis articles focused on examining complex systems (4/4) and identifying interventions (4/4), they did not focus on PA (0/4) nor use participatory methods (0/4). All attributes were discussed in some way in the 25 articles (Table 2). Attributes were either explicitly reported on in terms of findings (less so) or were part of the discussion and conclusion sections (more so). System mapping methods appear to be well aligned with a whole of system approach because these methods addressed all attributes in some way. We did not find this pattern in other methods. Brennan et al used group model building and they discussed all attributes: *Complex System Paradigm* (e.g., identify causal relationships among variables), *Implementation of Desired Actions* (e.g., health behaviors, active living policies and environments); *Collaborative Capacity* (e.g., partnerships, community/civic engagement, social ties); *Resources* (knowledge and skill, financial/in kind resources); *Information* (research and evaluation); *Leadership* (political will, community leadership); *Health Equity Paradigm* (e.g., social determinants of economy, employment, public transportation, targeted support to poor families, access to opportunities, neighborhood associations). Articles that reported on simulation modeling methods appeared to align most with discussions of *Implementation of Desired Actions* and network analysis with *Collaborative Capacity*. The following provides examples of how attributes and associated dimensions were discussed. |
| Limitations | 20 | Discuss the limitations of the scoping review process. | We found 25 articles that met our inclusion criteria and these articles provided a broad overview of complex systems methods used in population physical activity research and the extent to which they align with attributes of a whole of system approach complex systems methods used on PA research. Limitations include a search strategy targeted to two databases and English only articles. These factors, along with no grey literature, may have limited our findings. Furthermore, some articles were excluded because they did not explicitly use a complex systems research method but used concepts in analysis or discussed theory. These articles may contain new insights for taking a whole of systems approach. Finally, identifying and reporting on leverage points (i.e., interactions among attributes in Table 2) was based solely upon our interpretation of findings, discussion and conclusions in the articles. This was clearly exploratory in nature and authors could well place different emphasis on their articles. |
| Conclusions | 21 | Provide a general interpretation of the results with respect to the review questions and objectives, as well as potential implications and/or next steps. | We found that system mapping methods were most aligned with a participatory whole of system approach and also that the Attributes Model was validated to some extent. Taken together these key findings suggest future PA research could apply the model in conjunction with system mapping methods. Furthermore, simulation modeling and network analysis methods were seen as complementary and could be used when system mapping methods identify priorities for further investigation (e.g., what interventions to implement or how densely connected are relationships in systems). In conclusion, we suggest that this model and these methods help to lift our collective gaze to whole systems and offer an understandable approach for PA research, policy and practice. |
| **FUNDING** | | | |
| Funding | 22 | Describe sources of funding for the included sources of evidence, as well as sources of funding for the scoping review. Describe the role of the funders of the scoping review. | This research was supported by a Health System Impact Fellowship for LBL from the Canadian Institute for Health Research, Michael Smith Health Research BC and the BC Centre for Disease Control. |

JBI = Joanna Briggs Institute; PRISMA-ScR = Preferred Reporting Items for Systematic reviews and Meta-Analyses extension for Scoping Reviews.

* Where *sources of evidence* (see second footnote) are compiled from, such as bibliographic databases, social media platforms, and Web sites.

† A more inclusive/heterogeneous term used to account for the different types of evidence or data sources (e.g., quantitative and/or qualitative research, expert opinion, and policy documents) that may be eligible in a scoping review as opposed to only studies. This is not to be confused with *information sources* (see first footnote).

‡ The frameworks by Arksey and O’Malley (6) and Levac and colleagues (7) and the JBI guidance (4, 5) refer to the process of data extraction in a scoping review as data charting*.*

§ The process of systematically examining research evidence to assess its validity, results, and relevance before using it to inform a decision. This term is used for items 12 and 19 instead of "risk of bias" (which is more applicable to systematic reviews of interventions) to include and acknowledge the various sources of evidence that may be used in a scoping review (e.g., quantitative and/or qualitative research, expert opinion, and policy document).

## File S2. Search strategy

Ovid MEDLINE(R) and Epub Ahead of Print, In-Process, In-Data-Review & Other Non-Indexed Citations, Daily and Versions(R) and Web of Science databases were searched using the follow terms:

complex systems.mp.
system* dynamics.mp.
1 or 2
Exercise/
Physical Fitness/
(physical activity or sedentary or physical fitness).mp.
(complex adj2 systems).mp.
4 or 5 or 6
2 or 7
8 and 9
(systems science or systems thinking or systems practice).mp.
2 or 7 or 11
8 and 12
limit 13 to (english language and yr="2010 -Current")
health promotion/ or healthy people programs/
health promotion.mp.
chronic disease prevention.mp.
15 or 16 or 17
8 or 18
12 and 19
limit 20 to (english language and yr="2010 -Current")

## File S3. Detailed summary of topics and key findings

Overall, key findings provided a broad spectrum of actions to address specific topics with respect to promoting PA (Table 3). *Action* is a catch all term used here to categorize PA policies, programs, strategies, regulations, and laws. Addressing actions was congruent with the general direction of most research aims, that is, to inform policy and practice with respect to PA promotion. Only one article did not specifically report on an ‘action’ as defined here (46) and is discussed below.

Active transportation

Five articles explicitly reported on active transportation. Yang et al (42) found through simulation modeling (system dynamic modeling) that economic development and urban sprawl are likely more influential than urban design and crime in terms of influence on active transportation to school and this was an inverse relationship. Another example of system dynamics modeling of active transportation is provided by McMillan et al (38). Here they report that the greatest impact on active transportation are polices and practices to physically segregate arterial roads, to lower speed, and to make streets bicycle friendly. Salvo et al (37) used agent-based modeling and determined that at-scale actions that included transport systems that prioritize walking, cycling, and transit and activity-promoting urban design could provide benefits. Using group modeling building, Brennan et al (23) found that a common variable with respect to active living policies was active transportation (e.g., access to public transportation, complete streets). Finally, two main domains of physical activity were identified as walking for transport and cycling for transport through conceptual mapping (30).

Built environment and urban design

Beyond articles that specifically discussed active transportation, many others reported on broad actions to enhance the built environment, physical infrastructure, and/or urban design (13, 25, 26, 28, 29, 31, 32, 36, 44, 45, 47). For example, Murphy et al (13) mapped current policy and practice to the WHO’s Global Action Plan on Physical Activity and found that strengthening policy, regulatory, and design guidelines for physical activity engagement in and around public buildings and public places, and the improvement of walking and cycling infrastructure would be impactful. Carlson et al (29) used a causal pathways diagram and found the strongest associations with PA were with respect to destination walking, sidewalks and connectivity and Signal et al (32) used a system map to also find that connectivity as well as improving urban design of open space were key findings. Physical activity infrastructure policy was found to have the greatest impact on the reduction in the BMI Disparity Index in an agent-based modeling study conducted by Orr et al (36). As a final example, Blackford et al (45) found through social network analysis (and a system inventory of actions) that the least common organizational objectives were changes to the built environment, regulations and advocacy.

Although some articles did not report built environment, physical infrastructure, and/or urban design as a key finding, they offered some important insights. For example, Garcia et al (44) used agent-based modeling and found that psychological attributes were "the strongest proximal determinants of LTPA [leisure time physical activity], however, this relationship is dynamically moderated by the built environment and influenced by both the social environment and the behavior itself" (p9).

Safety and the physical environment

Another topic addressed in the articles was safety and the physical environment. Three articles using group model building discussed this topic. Guariguata et al (26) reported that there was ample space for physical activity but these were not always well-maintained, safe or accessible to the public. This was echoed by Keane et al (24) who found that more safe places to be physically active was linked to increased activity levels. A circular pattern was found with respect to the social norms towards walking and cycling, perceived safety, and walking and cycling behaviour by Waterlander et al (27).

Following on this, there were three articles that addressed safety and PA through simulation modeling (Table 1). High street safety from crime in conjunction with low car use, high public transportation subsidies, and more free time were associated with favorable health outcomes by Stankov et al (43) using cross impact analysis. Similarly, Yang et al (42) modeled increased crime combined with economic development, urban sprawl, and poor urban design decreases active transportation to school. A final example addressed the issue of injury. Like Yang et al (42), MacMillan et al (39) used system dynamic modeling and reported that "Although our findings suggest that Auckland’s existing plan to develop a regional cycle network would likely have benefits, the simulation modeling suggests that it would not reverse the predicted business-as-usual increased rate of cycling injury. In contrast, a gradual transformation of all roads using best practice arterial and local street interventions could make a major contribution to regional transport targets"(p342).

Social Environment

Although discussion of the social environment was woven throughout the articles, fewer articles explicitly discussed its role in PA. Bellew et al (28) identified social environment and norms as one factor among many that influences PA in their conceptual system map and Frerichs et al’s (35) agent-based modeling study showed that social interactions were likely to influence physical activity choices. A final example is from articles using group model building methods where cultural norms were found to discourage physical activity (i.e., negativity towards sweating which influences active transport and this was associated with low socioeconomic status) and these cultural norms were stronger for women (26).

Schools

School settings were either the focal point or found in research results in seven articles (23, 24, 35, 37, 40, 41, 42, 37). For example, both Soler et al (41) and Powell et al (40) used system dynamics modelling and found respectively, the greatest impact of obesity interventions were found to be a) increased physical activity in schools and child-care facilities and b) daily physical education at school and the integration of moderate to vigorous physical activity into elementary school classrooms. Another example from Keane et al (24) described three key findings: excessive time on the school bus was linked to inactivity, after-school buses allowed for extracurricular physical activities, and a curriculum that blends academics and physical activity was needed. The latter was reported from using group model building (causal loop diagrams and behaviour over time graphs).

Children

Beyond articles centered on school settings, two articles reported on research about children’s PA. Children's physical activity or active living behaviors (i.e., walking to school, TV time, play outside) were found exhibit decreasing trend lines in behaviour over time graphs (25). Almagor et al (34) used agent-based modeling and found that neighborhoods can enhance the engagement of children in physical activity and encouraging children to be active in diverse groups would likely have a positive effect on the least active. They further reported that the most important characteristic in influencing PA levels was found to be the agent’s tendency to be active and the second most important factor was the walking time of the agent.

Socio-ecological perspectives

Several articles focused on the individual level of analysis, however, there were calls to move beyond this. For example, using agent-based modeling, Garcia et al (38) identified three elements that more strongly influenced time trends of people practicing leisure-time physical activity (LTPA) and these were: “the influence of the person’s behavior in the previous week over his current intention, size of the person’s perception radius, and proportion of LTPA sites in the model" (p9). Some articles addressed a broader perspective. For example, using concept mapping, Holdsworth et al (31) found that psychosocial factors that influence physical activity behaviours of ethnic minority populations was the highest ranked cluster in terms of priority for PPA research and intervention. However, psychosocial factors were discussed in relation to institutional, political, social and cultural, and physical environments, social and material resources, health communication, and migration contexts. Cavill et al’s (30) work from conceptual mapping reported that most actions to promote PPA were focused at the interpersonal level and Blackford et al (45) reported a similar finding from social network analysis. Here they found that the organizational objectives they studied targeted individual behaviour change, knowledge, skills, and awareness. A final example from Waterlander et al (27) stated that system change was needed in areas such as macroeconomics, social welfare, technology, and urban systems rather than a focus on actions targeting individual behaviour change.

Sports

It is notable that few articles explicitly discussed sports. Of those that did, Cavill et al (30) identified sport and active recreation as one of three specific domains of physical activity (including walking for transport and cycling for transport) in their conceptual map. Sports-for-all programs were identified by Almagor et al (34) and Salvo et al (37) and both used agent-based modeling.
